# Senescence of Primary Amniotic Cells via Oxidative DNA Damage

**DOI:** 10.1371/journal.pone.0083416

**Published:** 2013-12-27

**Authors:** Ramkumar Menon, Istvan Boldogh, Rheanna Urrabaz-Garza, Jossimara Polettini, Tariq Ali Syed, George R. Saade, John Papaconstantinou, Robert N. Taylor

**Affiliations:** 1 Department of Obstetrics and Gynecology, Division of Maternal-Fetal Medicine Perinatal Research, The University of Texas Medical Branch at Galveston, Galveston, Texas, United States of America; 2 Department of Microbiology and Immunology, The University of Texas Medical Branch at Galveston, Galveston, Texas, United States of America; 3 Department of Biochemistry and Molecular Biology, The University of Texas Medical Branch at Galveston, Galveston, Texas, United States of America; 4 Department of Obstetrics and Gynecology, Wake Forest School of Medicine, Winston-Salem, North Carolina, United States of America; John Hunter Hospital, Australia

## Abstract

**Objective:**

Oxidative stress is a postulated etiology of spontaneous preterm birth (PTB) and preterm prelabor rupture of the membranes (pPROM); however, the precise mechanistic role of reactive oxygen species (ROS) in these complications is unclear. The objective of this study is to examine impact of a water soluble cigarette smoke extract (wsCSE), a predicted cause of pregnancy complications, on human amnion epithelial cells.

**Methods:**

Amnion cells isolated from fetal membranes were exposed to wsCSE prepared in cell culture medium and changes in ROS levels, DNA base and strand damage was determined by using 2′7′-dichlorodihydro-fluorescein and comet assays as well as Fragment Length Analysis using Repair Enzymes (FLARE) assays, respectively. Western blot analyses were used to determine the changes in mass and post-translational modification of apoptosis signal-regulating kinase (ASK1), phospho-p38 (P-p38 MAPK), and p19^arf^. Expression of senescence-associated β-galectosidase (SAβ-gal) was used to confirm cell ageing *in situ*.

**Results:**

ROS levels in wsCSE-exposed amnion cells increased rapidly (within 2 min) and significantly (p<0.01) at all-time points, and DNA strand and base damage was evidenced by comet and FLARE assays. Activation of ASK1, P-p38 MAPK and p19^Arf^ correlated with percentage of SAβ-gal expressing cells after wsCSE treatment. The antioxidant N-acetyl-L-cysteine (NAC) prevented ROS-induced DNA damage and phosphorylation of p38 MAPK, whereas activation of ASK1 and increased expression of p19^Arf^ were not significantly affected by NAC.

**Conclusions:**

The findings support the hypothesis that compounds in wsCSE induces amnion cell senescence via a mechanism involving ROS and DNA damage. Both pathways may contribute to PTB and pPROM. Our results imply that antioxidant interventions that control ROS may interrupt pathways leading to pPROM and other causes of PTB.

## Introduction

Intrauterine oxidative stress during pregnancy is a natural physiologic response to fetoplacental energy demand [1], [2]. Generation of reactive oxygen species (ROS) is an intrinsic and inevitable result of aerobic energetic, but the process is well balanced in healthy pregnancy by a combination of enzymatic and non-enzymatic antioxidant redox systems [3]–[5]. Imbalanced redox status is a feature underlying many pregnancy complications [6], particularly spontaneous preterm birth (PTB) and preterm premature rupture of the membranes (pPROM), and is associated with increased oxidative stress [7]–[9]. Risk factors for PTB and pPROM, including cigarette smoking, infection, poor nutrition, and obesity are associated with oxidative stress (superoxide anion, hydrogen peroxide, hydroxyl radicals and nitric oxide generation) that damage the pericellular collagen matrix and consume antioxidant defenses [10]–[12]. Overwhelming placental ROS production is thought to lead to inflammation and other “initiators” of PTB and pPROM, but their mechanisms of action remain unclear.

Recently we demonstrated that women who smoked cigarettes during pregnancy had elevated levels of amniotic fluid F2-Isoprostane (F2-IsoP), an established marker of oxidative stress, relative to normal pregnant controls and even women with intraamniotic infection. This finding suggests that the degree of ROS production might predict specific pregnancy complication risks and pathways [13].

ROS generated by environmental insults or endogenously during pregnancy can oxidize proteins, lipids and nucleic acids [2], [14]. F2-IsoP and placental telomere shortening (as we have shown in pPROM reflect lipid and DNA peroxidation damage by ROS, respectively [13], [15]. A recent report showed that even passive cigarette smoking is associated with fetal DNA lesions, due in part to impaired DNA damage repair mechanisms [16]. Oxidized DNA base adducts such as the highly mutagenic 8-oxo-7, 8-dihydroguanine (8-oxoG) lesion, is predominantly repaired via the base excision repair pathway by 8-oxoguanine DNA glycosylase (OGG1 [17], [18]. Failure to repair these nucleoside lesions leads to DNA strand breaks and loss of genomic integrity [19], [20]. When these accumulate in guanine-rich telomere sequences they can result in telomere-initiated senescence [19]–[25]. Besides telomeres, unrepaired 8-oxoG in the genome is linked to other ageing related pathologies. Moreover, a recent report by Boldogh et al showed that cellular signaling activated by OGG1 [26] activates inflammatory responses similar to those documented in pPROM.

One of the primary effectors of ROS-induced senescence is the p38 mitogen activated protein kinase (p38 MAPK) pathway [27]. p38 MAPK activity induces programmed cell death via the apoptosis signal-regulating kinase (ASK1)-signalosome [28], [29]. ROS-mediated oxidation of ASK1 activates the p38 MAPK and its downstream effectors, phospho-p38 MAPK (P-p38 MAPK), p16^Ink4^ and p19^arf^, resulting in cell cycle arrest and senescence. Furthermore, studies by Hsieh et al have shown that ROS generated by dysfunctional electron transport in mitochondria activate the inflammatory Ask1-P-p38 MAPK pathway [28], [30].

To test our postulation that DNA damage and fetal membrane senescence may constitute mechanistic pathways of PTB and pPROM, we interrogated normal amnion epithelial cells with water soluble cigarette smoke extract (wsCSE) [31] by measuring ROS-induced DNA base (8-oxoG) and strand damage, as well as signaling intermediates of premature cellular senescence.

## Materials and Methods

Placental samples for this study were obtained from subjects who delivered at John Sealy Hospital, The University of Texas Medical Branch (UTMB) at Galveston, TX, USA. Institutional Review Board at UTMB has approved this study (protocol number 11–251) and waived the requirement for obtaining informed written consent from subjects for this study as we were using discarded placental samples.

### Amnion Cell Culture

Primary amnion epithelial cells (n = 8) were isolated as previously described from placentas from normal parturient at term and not in labor undergoing repeat elective Cesarean sections [32]–[35]. Briefly, reflected amnion (about 10 g), was peeled from the chorion laeve and dispersed by successive treatments with 0.024% collagenase and 1.2% trypsin. The dispersed cells were allowed to sediment at unit gravity force and were plated in a 1∶1 mixture of Ham’s F12/DMEM, supplemented with 20% heat-inactivated fetal bovine serum (FBS), 10 ng/ml EGF, 2 mM L-glutamine, 100 U/ml penicillin G and 100 µg/ml streptomycin at a density of 3×10^6^ cells per well in 6-well plates to yield cultures with 95–99% purity. Viability of cells was tested using Trypan blue exclusion. The epithelial nature of the primary cell cultures was verified by immunocytochemistry using anti-human cytokeratin antibodies as described by Moore et al [35] and all our cultures had >95% cytokeratin positive cells.

### Preparation of wsCSE

wsCSE was prepared by bubbling smoke drawn from a single lit commercial cigarette (unfiltered Camel™, R.J. Reynolds Tobacco Co, Winston Salem, NC) through 50 ml of tissue culture medium (Ham’s F12/DMEM mixture with antimicrobial agents and filter sterilized through a 0.22 µm Millipore filter (Bedford, MA) to remove contaminant microbes and insoluble particles [31]. Amnion cells were stimulated with 1∶10 dilutions of wsCSE in culture media by incubation at 37C for up to 6 hours. The media were removed and frozen for subsequent for analysis.

### Measurement of ROS

Amnion cells grown to 70% confluence were loaded with 50 µM 2′7′-dichlorodihydro-fluorescein (H_2_DCF) diacetate at 37°C for 30 minutes and cells were exposed to wsCSE and/or the antioxidant N-acetyl cysteine (NAC; 10 µM) for up to 6 hours. To determine changes in ROS levels, fluorometeric measurements were taken after 2 min and every 15 min for the first hour and at 1 hr intervals for a 6 hr period. DCF fluorescence was recorded at 528 nm after excitation at 485 nm in an FLx800 microplate reader. Results are expressed as arbitrary units, calculated using the mean slope of a linear regression of all points within the calculation zone.

### Comet Assay

The assay was performed as previously reported [36], [37] using reagents from Trevigen Inc. (Gaithersburg, MD) according to the manufacturer’s instructions. wsCSE treated amnion cells were embedded in a layer of low melting point agarose and transferred to Trevigen-slides at 37°C. Electrophoresis was conducted for 30 min at 21 V. Fifty cells per culture were counted under an Olympus microscope (40×objectives) and scoring of the comet tail DNA content was performed using the Comet Assay IV v4.2 system (Perceptive Instruments, Suffolk, UK). The control (untreated) cells were used to establish the normal DNA content of a healthy cell with nominal comet formation.

### FLARE® (Fragment Length Analysis Using Repair Enzymes) Assay

The FLARE modification of the comet assay above was conducted using Trevigen reagents according to the manufacturer’s protocols. Electrophoresed wsCSE-treated amnion cells on agarose slides were immersed in lysis solution (2.5 M NaCl, 100 mM EDTA pH 10, 10 mM Tris base, 1% sodium lauryl sarcosinate, 1% Triton X-100 and 1% DMSO) for 1 h at 4°C and washed in FLARE buffer (250 mM HEPES-KOH pH 7.4, 2.5 M KCl and 250 mM EDTA) at room temperature three times over a 15 min period and OGG1 protein was added in a digestion buffer (FLARE buffer) incubated at 37°C for 40 min. The DNA in the agarose gels was denatured in electrophoresis buffer pH 12.1 (3 M NaCl, 500 mM EDTA) for 30 min at 4°C and separated by electrophoresis in alkaline solution (pH 13) at 300 mA, 25 V for 30 min at 4°C. The slides were placed in cold methanol for 20 min, dried and stored in a slide box at room temperature. At time of analysis, the slides were hydrated in cold water at 4°C for 20 min and stained with ethidium bromide solution (2 µg/mL). Like the comet assay, the amount DNA in the FLARE tails was quantitated as above.

### Western Blot Analysis

Amnion cells were homogenized in RIPA buffer with protease inhibitors using a bullet blender (Next Advance, Averill Park, NY). Protein quantification was done using the Pierce BCA protein assay kit (Thermo Scientific, Rockford, IL). Samples containing 45 µg of protein were separated by SDS-gel electrophoresis (Bio-Rad, Hercules, CA) according to manufacturer’s suggestions and proteins were transferred to a PVDF membrane using the iBlot dry blotting system (Life Technologies, Grand Island, NY). The membranes were blocked for two hours in 5% milk in TBS-Tween-20. The blots were then incubated with primary antibody to ASK1 (Abcam), total p38 (Cell Signaling #9212, Danvers, MA), P-p38 (Cell Signaling #9211, Danvers, MA) or p19^arf^ (Santa Cruz Biotechnologies, Inc., Dallas, TX) overnight at dilutions of 1∶800, 1∶1,000, 1∶400 and 1∶200, respectively. Blots were then washed and incubated with secondary antibody for 1 hour and revealed with Pierce ECL2 chemiluminescence detection reagent (Thermo scientific #8019). In order to avoid inter-assay variability between blots, samples from the same experiments were run on the same gel for a given marker. The blots were all reprobed with antibodies to β-actin (Sigma, St. Louis, MO) Detected bands were then analyzed densitometrically using the Image J software (National Institutes of Health, rsbweb.nih.gov/ij) and results were normalized to β-actin expression on the same blots. P-p38 were also normalized with total p38 values.

### Senescence by Senescence-associated β-galectosidase Assay (Saβ-gal)

The expression of the SAβ-gal biomarker is independent of DNA synthesis and distinguishes senescent from quiescent cells [38]. This enzymatic activity is distinct from the ubiquitous acidic β-galectosidase and can be detected at pH 6.0 with the chromogenic substrate X-gal. Senescent cells were identified using a histochemical staining kit (Sigma, St. Louis, MO) with blue cells visualized by light microscopy 3 hours after treatment with wsCSE. The proportion of positive cells in the total cell population was counted manually and reported for wsCSE-treated and untreated cultures.

### Statistical Analysis

For the quantitative Western data analysis, we used a repeated measures two-way ANOVA, considering treatment and time factors as the variables. Tukey’s multiple comparisons test were performed to correct for pair wise treatment effects. All data were analyzed using GraphPad Prism 6 for Windows.

## Results

### Water Soluble CSE Induces ROS in Amnion Epithelial Cells

**Figure 1:**
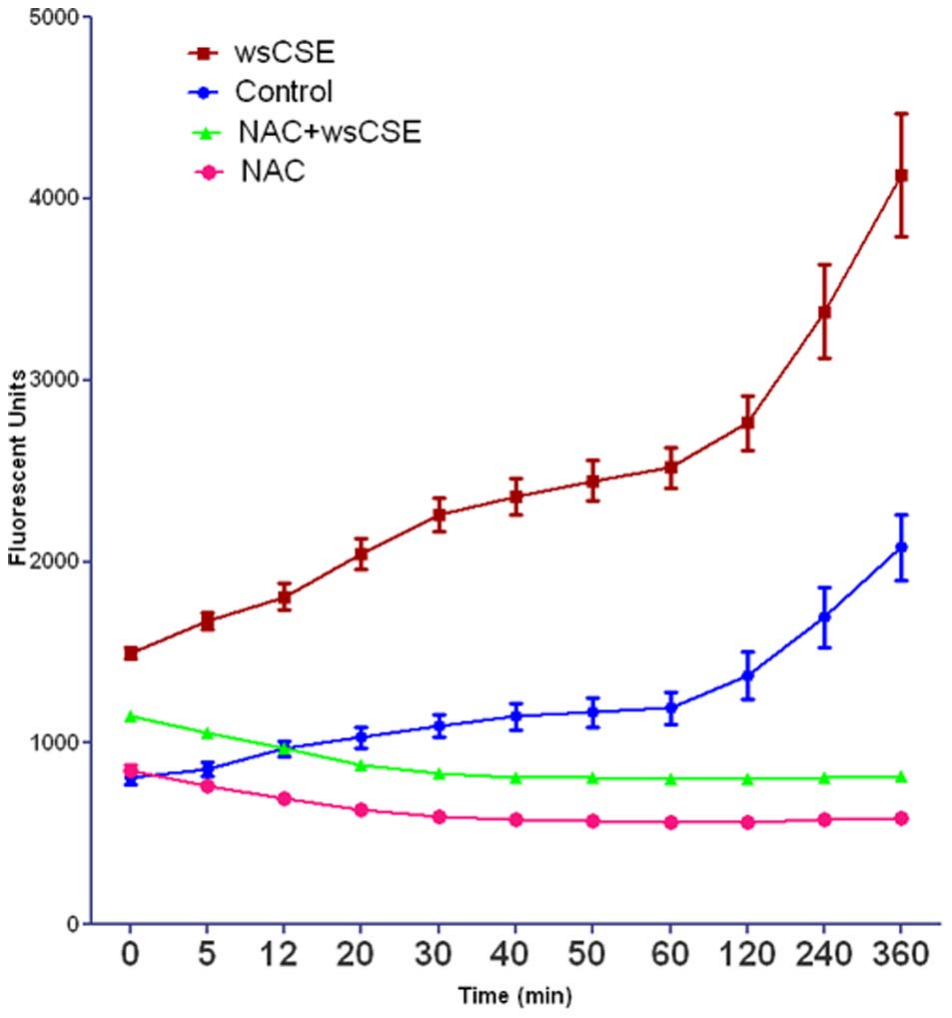
wsCSE induces increased ROS levels in primary amnion cells. Preincubation with the antioxidant N-acetyl cysteine (NAC) prevented ROS accumulation (n = 8). Controls – Untreated amnion cells in culture. Data were significant (p<0.05) for all time points for wsCSE treated cells compared to both control and wsCSE+NAC treated amnion cells.

Amnion cells treated with wsCSE showed increased ROS levels within 2 minutes of exposure that were significantly higher than untreated controls (p<0.05 for all time points) (Figure 1). However, treatment with wsCSE in the presence of NAC prevented the increase in ROS levels and in fact, reduced ROS below levels of control cells (p<0.05 for all time points). Although site of ROS generation yet to be determined these data imply that amnion cells rapidly respond to wsCSE.

### DNA Damage in wsCSE-exposed Amnion Cells

**Figure 2:**
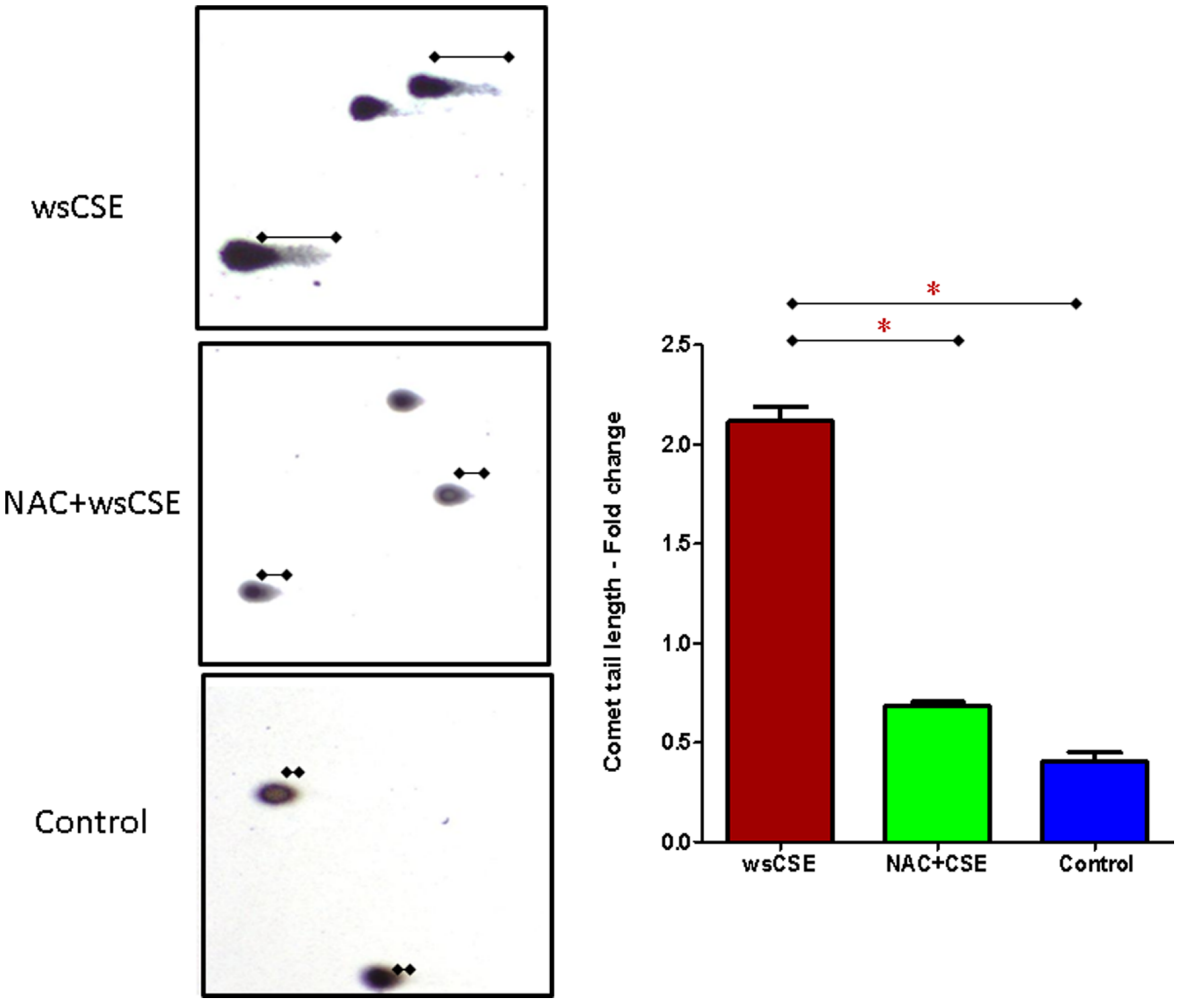
wsCSE induces DNA base and strand damage. DNA damage was determined by comet assays (n = 4) as described in the text. DNA damage was prevented by treatment with NAC prior to CSE exposure. Comet tail lengths were determined microscopically. Untreated amnion cells (control) had minimal comet or FLARE formation. *indicates significant differences (p<0.05).

**Figure 3:**
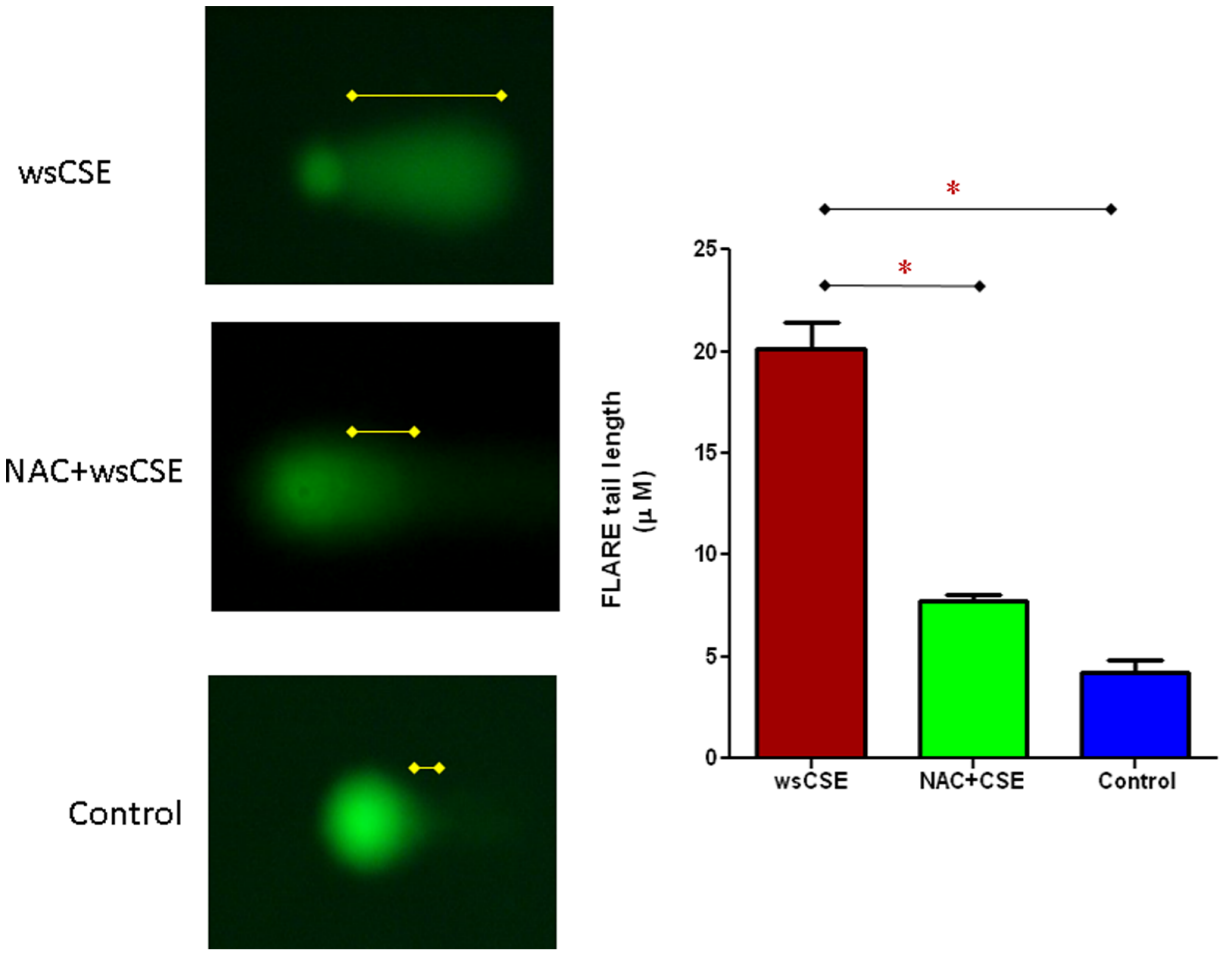
wsCSE induces DNA base and strand damage. DNA damage was determined by FLARE assays (n = 4) as described in the text. DNA damage was prevented by treatment with NAC prior to wsCSE exposure. FLARE tail lengths were determined microscopically. Untreated amnion cells (control) had minimal FLARE formation. *indicates significant differences (p<0.05).

Both the comet and FLARE assays revealed that wsCSE induced DNA damage in amnion cells. The comet assay showed that 3 hours exposure to wsCSE induced ∼5-fold more DNA strand breaks compared to levels in unstimulated control cells (p<0.05). Pre-treatment of cells with NAC prevented DNA strand damage by ∼60% (p<0.05) (Figure 2). Changes in oxidized nucleoside, 8-oxoG, upon wsCSE exposure were estimated by FLARE assays (Figure 3). Results revealed a >5-fold increase in the level of oxidized guanine substrates of OGG1 (8-oxoG and FapyG) after treatment with wsCSE 20.12 µM ±2.295 µM vs. 4.23 µM ±1.432 µM; p<0.01). Our study also showed that the wsCSE treatment-induced base damage was decreased to 7.74 µM ±0.71 µM (p<0.01) in cells treated with NAC. Untreated control cells had minimal levels of FLARE tails. Although direct nucleoside damage by toxic chemicals in wsCSE cannot be excluded, our results are consistent with ROS-induced lesions that are generated directly within the cultures and can be perpetuated as intermediates during DNA repair.

### Increased ASK1, P-p38 MAPK and p19^arf^ in Amnion Cells

**Figure 4:**
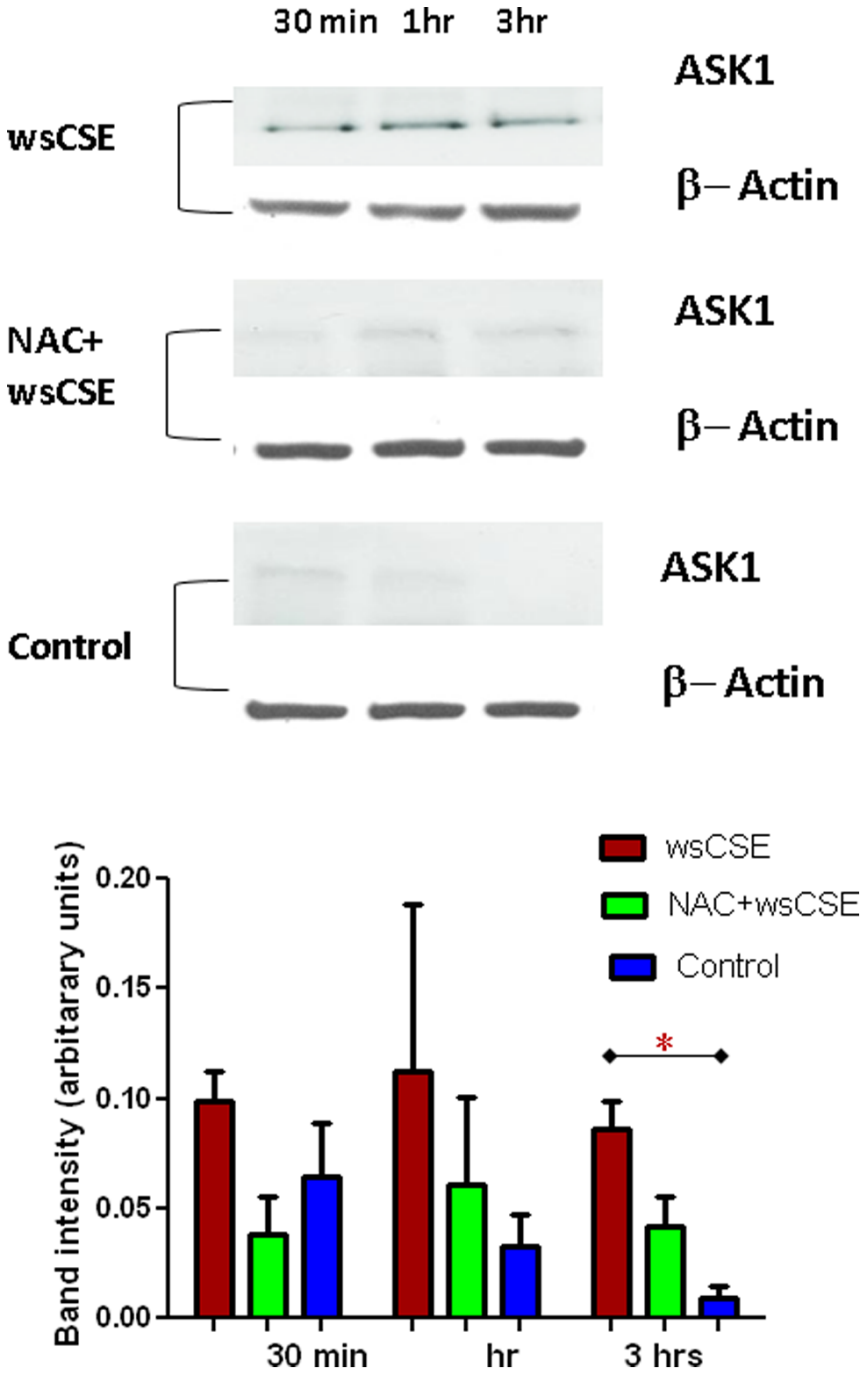
wsCSE increases ASK1 protein levels. ASK1 (155 kDa) and β-actin (42 kDa) protein bands were identified in amnion cells. Bar graphs demonstrate densitometric quantitation of Western blot band densities (n = 4) normalized to β-actin levels. *indicates significant differences (p<0.05).

**Figure 5:**
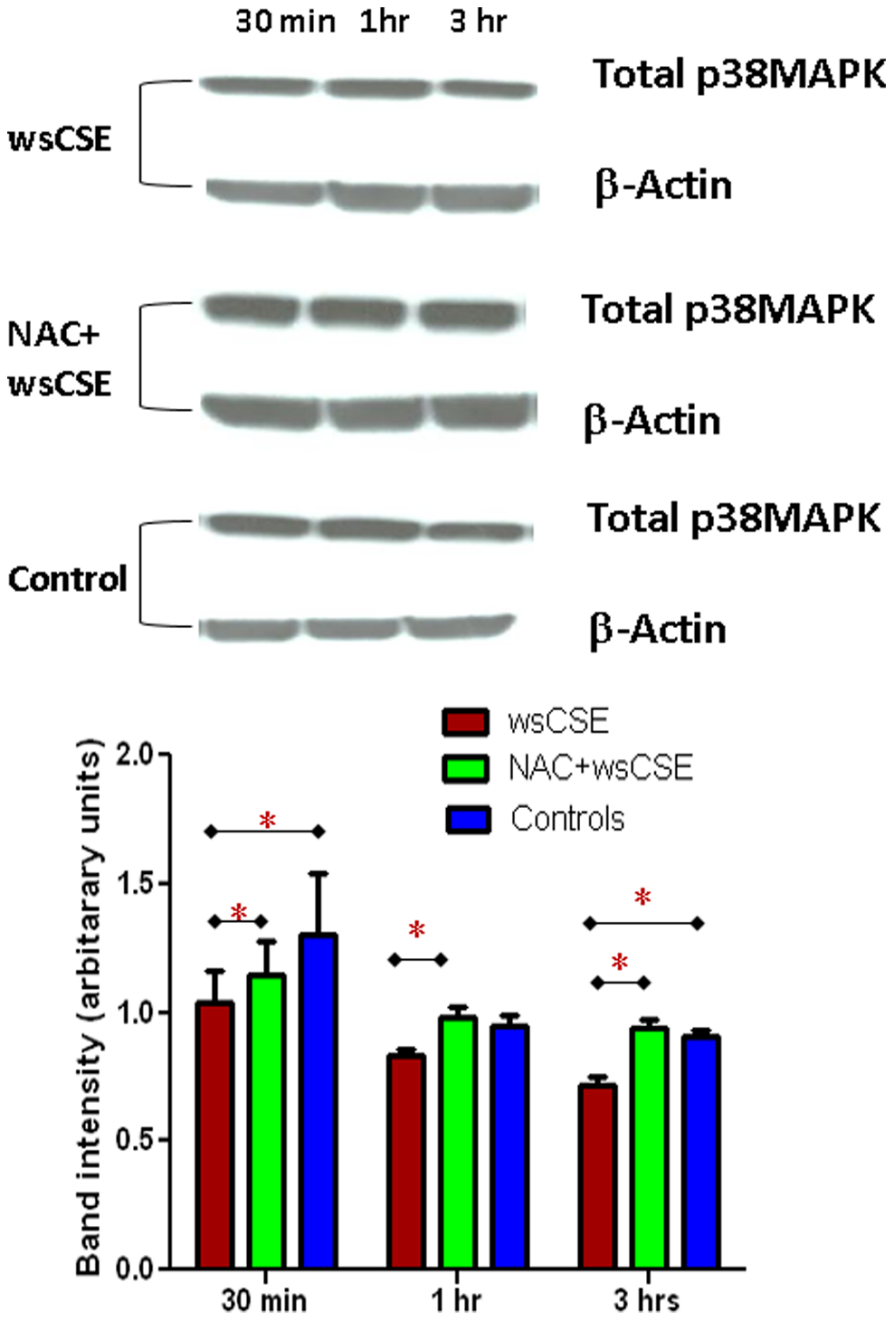
Western blot analysis of total p38 MAPK. Total p38MAPK (43 kDa) and β-actin (42 kDa) protein in amnion cells. Bar graphs demonstrate densitometric quantitation of band intensities normalized to β-actin (n = 4). *indicates significant differences (p<0.05).

**Figure 6:**
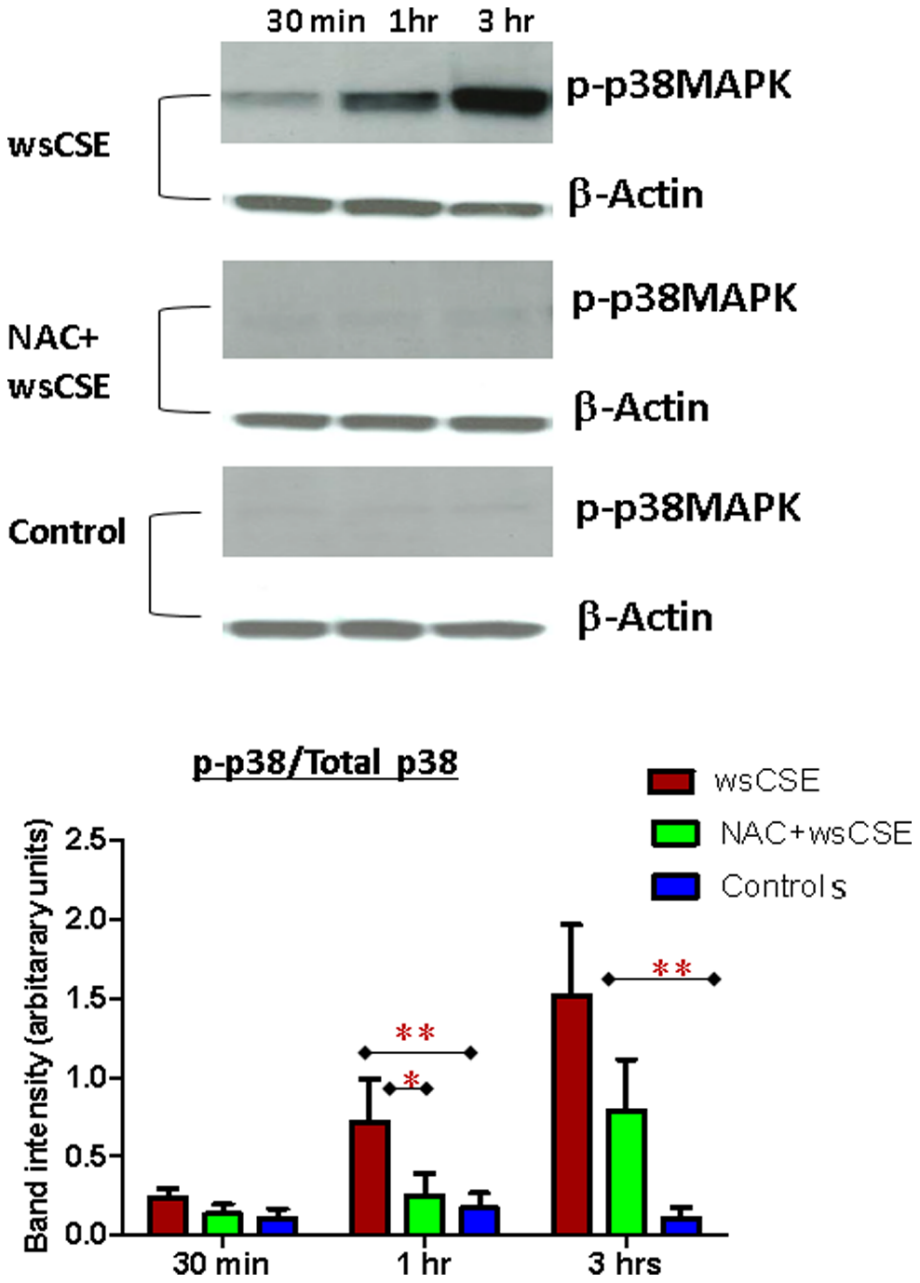
Western blot analysis of phosphorylated P-p38 MAPK. P-p38 MAPK (43 kDa) and β-actin (42 kDa) protein in amnion cells (n = 4). P-p38 MAPK showed a time dependent increase following wsCSE treatment that was significantly higher than in untreated cells at 1 and 3 hours. Treatment with NAC significantly decreased P-p38 at 1 and 3 hours. Bar graphs demonstrate densitometric quantitation of Western blot bands, normalized to total p-38 levels. **indicates significant p<0.01 and *indicates significant differences p<0.05.

**Figure 7:**
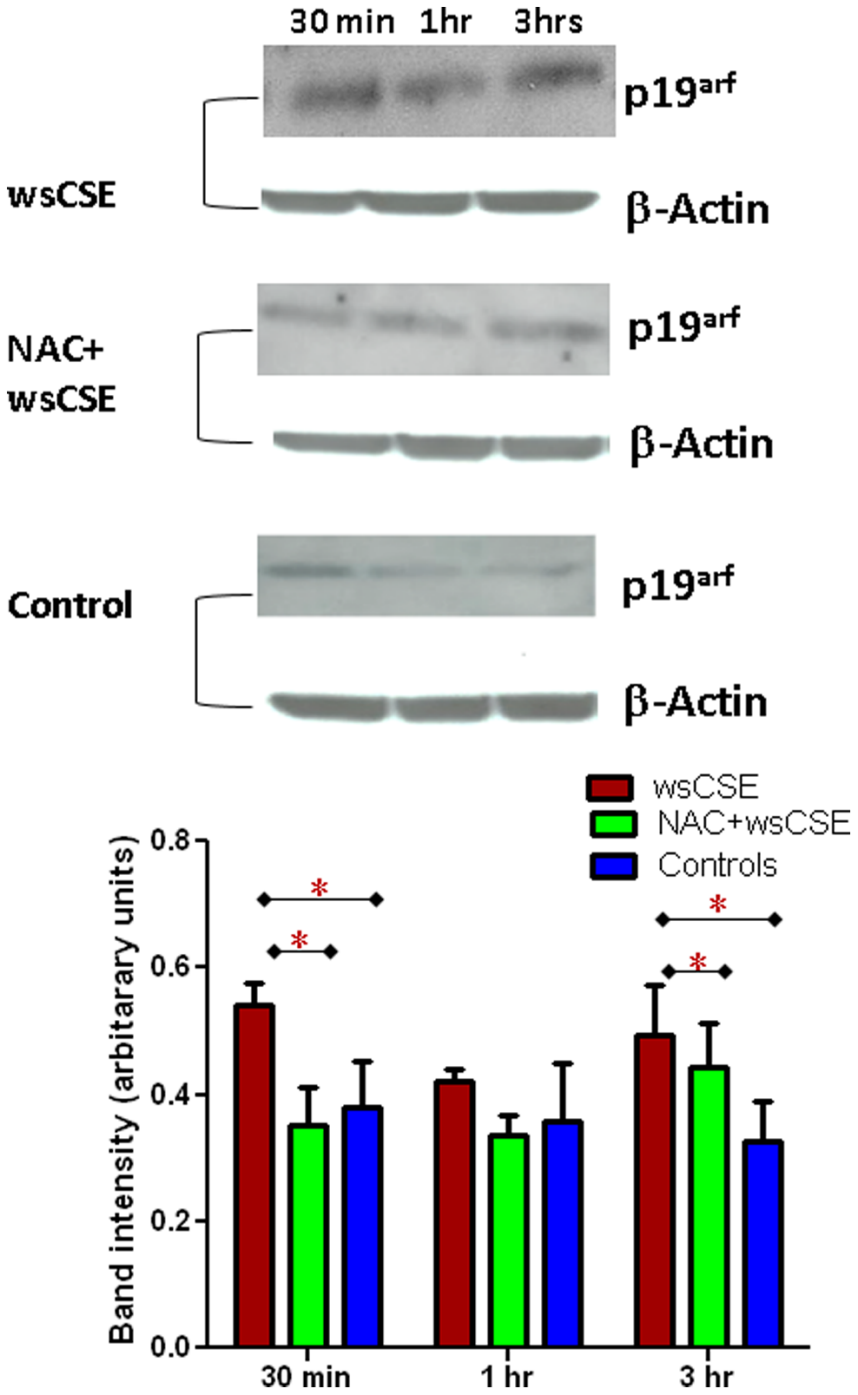
Changes in p19^arf^ in response to wsCSE: p19^arf^ (19 kDa) and β-actin (42 kDa) protein in amnion cells (n = 4). Co-treatment with NAC prevented changes in p19arf. Bar graphs demonstrate densitometric quantitation of Western blot bands, normalized to β-actin expression. *indicates significant differences (p<0.05).

Time course experiments were done to test whether ROS and DNA damage lead to increased expression of senescence related proteins in amnion cells. Western blots were performed and followed by densitometric quantitation of bands and normalization to β-actin signals. wsCSE-exposed amnion cells produced more ASK1, an activator of the p38 MAPK pathway (Figure 4) after 3 h, than control cells (p<0.05), although this effect was attenuated, it was not significantly prevented by NAC. Total p38 MAPK levels were lower in wsCSE-exposed relative to untreated controls at 30 mins and 3 hours (p<0.05), an effect that was reversed by NAC at 3 hours (Figure 5). Conversely, P-p38 MAPK was significantly higher in cells treated with wsCSE compared to unstimulated controls after 1 and 3 hours (Figure 6). Treatment with NAC restored the P-p38 MAPK response to control levels, confirming the ROS effect. The P-p38 MAPK mediator, p19^arf^, was also higher after wsCSE treatment compared to control at 30 min and at 3 hours (Figure 7) but NAC treatment had little effect on p19^arf^. In general, these markers support the hypothesis that ROS induce senescence in fetal amnion cells in response to wsCSE.

### Senescence Associated β-gal in wsCSE Exposed Amnion Cells

**Figure 8:**
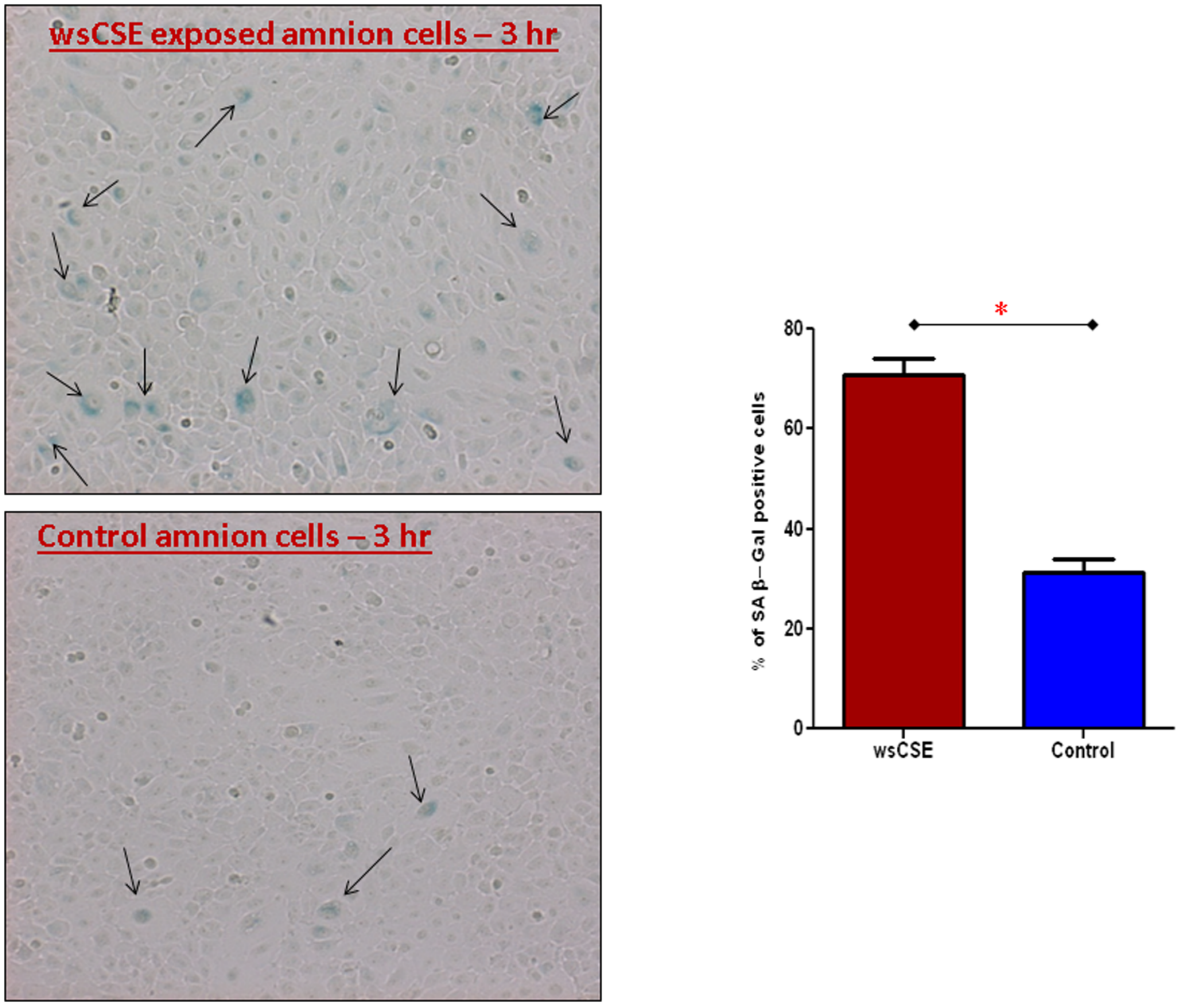
Senescence associated β-galactosidase amnion cells. Number of amnion cells stained for SAβ-Galactosidase was higher after wsCSE exposure compared to untreated controls (control) (n = 8). *indicates significant differences (p<0.05).

Amnion cells were grown to confluence and treated with or without wsCSE for 3 hours. The proportion of SAβ-gal-positive cells was higher after exposure compared to untreated controls (Figure 8). Results show a significant increase in percentage of cells stained for SAβ-gal after wsCSE treatment (71% vs. 31%; p<0.0001) compared to unstimulated controls. This result is consistent with the Western blot data showing increased ASK1, P-p38 MAPK and p19^arf^ accumulation after wsCSE treatment of cells.

## Discussion

The pathophysiologies of PTB and pPROM are complex [39], [40] and while overlapping, are not identical [41], [42]. Recent biomolecular and histologic data on pPROM and PTB suggest that increased ROS and oxidative damages to lipids and DNA in fetoplacental cells play an important pathophysiological role in these disorders. In the present study we show that wsCSE induces ROS in normal term amnion cells. We chose to test water soluble chemicals extracted from cigarette smoke as it has been well documented that these compounds circulate through the body fluids and impact organs beyond the respiratory tract [43]–[48]. Cigarette smoke contains over 7000 recognized chemicals [43], including nicotine, unsaturated aldehydes and heavy metals that are known inducers of ROS generation [44], [47] and DNA damage [48]–[50]. Cytotoxic and DNA damaging effects of environmental toxicants were reported in so-called “amnion-derived WISH cells” [51], [52], but the latter now are widely known to be identical to HeLa cells, presumably arising as a result of cell line contamination [53]. To our best knowledge, this is the first study to examine the effect of wsCSE on primary amnion epithelial cells. We document that as yet uncharacterized wsCSE components induce oxidative stress in amnion cells and cause DNA strand breaks by comet formation. The DNA lesion, 8-oxoG, was detected by digesting DNA with the OGG1 repair enzyme in FLARE assays. High 8-OxoG levels may explain the shortened telomere length we observed in a prior report [15] as these repetitive sequences are guanine rich and susceptible to ROS [54]. Although our study does not prove a direct link between DNA lesions and senescence, the association between telomere attrition and senescence has been confirmed elsewhere.

Concurrent activation of the ASK1-associated P-p38 MAPK pathway in amnion cells in response to wsCSE exposure also appears to be the effect of oxidative stress. The ASK1-signalosome, a signaling complex composed of several well-characterized proteins [55]–[57] can be oxidized by ROS, causing thioredoxin to dissociate from the complex and leading to the phosphorylation of p38 MAPK and its downstream effectors p16^ink4^ and p19^arf^. We observed coordinated ASK1, P-p38 MAPK and p19^Arf^ expression following wsCSE exposure, with a kinetic pattern consistent with oxidative stress. The amnion cells also exhibited a senescent phenotype, known to be a response to ASK1 activation, manifested by SAβ-gal staining. We propose that ROS signaling eventually leads to telomere shortening, cell cycle arrest and irreversible halt of cell proliferation.

Unlike apoptotic cells, senescent cells are retained in tissues and elicit inflammatory responses when encountered by innate immune cells. One relevant manifestation of this altered tissue environment is referred to as the senescence associated secretory phenotype (SASP), by which proinflammatory cytokines, chemokines, growth factors and matrix metalloproteinases are promulgated [27]. The same biomarkers are classically elevated in PTB and pPROM. We conclude from these studies that environmental factors such as cigarette smoke may induce PTB and pPROM via activation of SASP.

We believe that this is the first study to document that oxidative DNA damage induced in fetal membrane cells can lead to cellular senescence. The fact that water soluble factors derived from cigarette smoke can initiate this process *in vitro* supports an extensive epidemiological and clinical literature relevant to adverse pregnancy outcome. This novel pathway may thus explain and characterize a unique subset of complex PTB where redox imbalance plays an etiologic role. This is especially true in early pPROM and PTB<34 weeks, where oxidative stress and pronounced inflammatory conditions are present. Animal studies have shown that decidual senescence can lead to PTB by activating p53 (a proapoptotic factor) and inflammatory cytokines [58], [59].

A limitation of our study is the reliance upon an *in vitro* model of amnion epithelial dysfunction. However, the primary culture system we describe has been widely validated to recapitulate human amnion biochemistry and even tensile strength. One of the strength of this study is that it was designed to mimic the soluble toxicants in cigarette smoke, to which the fetal membranes would be subjected by way of the maternal circulation. Obviously, there are numerous factors in the wsCSE that potentially contribute to DNA damage and repair and induced SASP that we describe here, including a plausible temporal association of the ASK1 signalosome-p38 MAPK pathway. Our ongoing studies are designed to identify some of these mediators and clarify their possible interactions.

In summary, we have modeled one behavioral risk factor, cigarette smoking, but several others, including intraamniotic infections, alcohol and drug abuse, sexually transmitted infection, and poor nutrition are all associated with oxidative stress and PTB. We have demonstrated that wsCSE induces ROS that cause the following cascade: 1. DNA base and strand damage; 2) activation of the ASK-1 signalosome and P-p38 MAPK pathway; and 3) premature cellular senescence. The latter effect has been reported to lead to the SASP inflammation response and would be predicted to predispose to amniotic membrane fragility. Based on our observations we postulate that some cases of PTB, particularly those complicated by early pPROM, are likely to be disorders of fetal membrane redox status. Behaviors and nutritional factors outlined above are potentially reversible or preventable determinants of adverse fates of pregnancy. Interventions to mitigate ROS-induced damage provide attractive and tractable therapeutic objectives for the future.
